# Do outpatient statins and ACEIs/ARBs have synergistic effects in reducing the risk of pneumonia? A population-based case-control study

**DOI:** 10.1371/journal.pone.0199981

**Published:** 2018-06-28

**Authors:** Jiunn-Horng Kang, Li-Ting Kao, Herng-Ching Lin, Ta-Jung Wang, Tsung-Yeh Yang

**Affiliations:** 1 Department of Physical Medicine and Rehabilitation, Taipei Medical University Hospital, Taipei, Taiwan; 2 Department of Physical Medicine and Rehabilitation, School of Medicine, College of Medicine, Taipei Medical University, Taipei, Taiwan; 3 Graduate Institute of Life Science, National Defense Medical Center, Taipei, Taiwan; 4 Department of Pharmacy Practice, Tri-Service General Hospital, Taipei, Taiwan; 5 School of Health Care Administration, Taipei Medical University, Taipei, Taiwan; 6 Sleep Research Center, Taipei Medical University Hospital, Taipei, Taiwan; 7 Division of Cardiology, Department of Internal Medicine, Shuang Ho Hospital, Taipei Medical University, Taipei, Taiwan; University of Tampere, FINLAND

## Abstract

Whether statins and an angiotensin-converting enzyme inhibitors (ACEIs) / angiotensin receptor blockors (ARBs) are associated with reduced risks of infection events is still inconclusive. This study aimed to explore the risk of hospitalization for pneumonia among patients who had received treatment with ACEIs/ARBs and/or statins using a population-based dataset. This study included 19,281 patients as cases who were hospitalized for pneumonia and 19,281 controls. We used a logistic regression to compute the odds ratio (OR) and 95% confidence interval (CI) for having previously used statins or an ACEI/ ARB between patients who were hospitalized for pneumonia treatment and controls. We found there were significant associations between hospitalization for pneumonia and statin-only users (*p*<0.001), ACEI/ARB-only users (*p*<0.001), and statin and ACEI/ARB users (*p*<0.001). The logistic regression analysis suggested that statin-only users (adjusted OR = 0.38, 95% CI = 0.34~0.43), ACEI/ARB-only users (adjusted OR = 0.86, 95% CI = 0.82~0.91), and statin and ACEI/ARB users (adjusted OR = 0.47, 95% CI = 0.44~0.50) were all less likely to be hospitalized for pneumonia treatment than were non-users. Furthermore, we found that statin-only users (adjusted OR = 0.44, 95% CI = 0.40~0.50) and statin and ACEI/ARB users (adjusted OR = 0.55, 95% CI = 0.52~0.58) were less likely to be hospitalized for pneumonia treatment compared to ACEI-only users. However, combined statin and ACEI/ARB users (adjusted OR = 1.24, 95% CI = 1.10~1.40) were more likely to have been hospitalized for pneumonia treatment compared to statin-only users. Although we found use of both statins and ACEI/ARB were significantly associated with a lower risk of pneumonia, the combination of the two medications did not provide additional protection against pneumonia risk.

## Introduction

Angiotensin-converting enzyme inhibitors (ACEIs) / Angiotensin receptor blockers (ARBs) and statins are widely used to treat patients with cardiovascular diseases to modify primary and secondary risks. These medications have demonstrated beneficial effects in improving outcomes in patients with cardiovascular diseases [1,2]. Hypertension and hyperlipidemia are prevalent among cardiovascular patients; therefore, the combined use of these two kinds of medications is not uncommon in clinical scenarios. Some studies suggested that the combination of ACEIs/ARBs and statins may have synergic effects and produce better outcomes than their sole use in particular circumstances. For example, one study suggested that concomitant ACEI and statin use appeared to beneficially modulate changes in the glomerular filtration rate in men with stable coronary artery disease [3]. It was reported that a combination of and early treatment with statins and ACEIs/ARBs could prevent extensive inflammatory responses in patients undergoing coronary artery bypass surgery [4].

Pleiotropic effects of ACEIs/ARBs/statin have been demonstrated including improving endothelial and mitochondrial dysfunction, reduced reactive oxygen species, and modulating inflammatory cytokines [5,6]. Although mixed findings showed uncertain results regarding the beneficial effects of statins and ACEIs/ARBs on infectious events, statins and ACEIs/ARBs were suggested to primarily target on host responses to the pathogens [7], and have positive effects on outcomes of infectious diseases. ACEIs/ARBs exhibited anti-inflammatory and immunomodulatory effects in animal and human studies [8]. ACEIs/ARBs showed anti-inflammatory abilities in pulmonary and extrapulmonary sites [9]. In addition, ACEIs can increase the level of substance P and bradykinin in the upper airway and increase the coughing ability [10]. Therefore, ACEIs/ARBs were suggested to reduce the risk of pneumonia in elderly hypertension patients with Parkinson disease and in post-stroke patients with aspiration pneumonia [11–13]. One study also revealed that the use of ACEIs/ARBs was associated with a reduced risk of pneumonia in patients with chronic obstructive pulmonary disease (COPD) [14]. However, a well-designed randomized controlled trial in a general population that examines ACEIs/ARBs protecting against pneumonia is still lacking.

Similarly, the modulatory effect of statins on cytokine-mediated inflammatory responses may impact the prognoses of infectious diseases. Several reports suggested that statin treatment may also have beneficial effects in patients with pneumonia. One study reported that statins reduced the risk of hospitalization for pneumonia in myocardial infarction patients [15]. In a cohort study, statin use was not associated with reduced time until the first community-acquired pneumonia occurred in patients with COPD [16]. Although discordant results were exhibited among current studies, exploration of the effects against infectious diseases has garnered significant attention in clinical studies because of the easy availability of these medications and their applicability in the clinic. To our best knowledge, there are no data specifically analyzing possible synergic effects of the combination of ACEIs/ARBs and statins against infectious diseases.

The exposure timing of ACEI /ARBs/statins is important to clarify the mechanism and develop further clinical strategies in treating infectious diseases. In the present study, we explored the risk of hospitalization for pneumonia among patients who had received prior (outpatient) treatment with ACEIs/ARBs and/or statins using a large population-based retrospective case-controlled study. We utilized a nationwide medical registry to obtain adequate statistical power to estimate the risk of hospitalization for pneumonia.

## Methods

### Database

This case-control study used the Longitudinal Health Insurance Database (LHID2005), which is derived from the Taiwan National Health Insurance (NHI) program and is provided to scientists in Taiwan for research purposes. The LHID2005 includes medical claims of 1,000,000 enrollees who were systematically and randomly selected from the 2005 Registry of Beneficiaries (*n* = 25.68 million) of the Taiwan NHI program. The LHID2005 allows researchers to follow-up the medical utilization of these 1,000,000 enrollees since initiation of the Taiwan NHI program in 1995. Many researchers have employed data from the NHI program and published their studies in internationally peer-reviewed journals.

This study was approved by the Institutional Review Board of Taipei Medical University (TMU-JIRB N201704055) since the LHID2005 consists of de-identified secondary data released to the public for research purposes.

### Study sample

For cases, we first identified 54,240 patients who were hospitalized with a principal discharge diagnosis of pneumonia (ICD-9-CM codes 480~483.8, 485~486, and 487.0) from January 1, 2001 to December 31, 2013. If a patient had ≥ 2 hospitalizations for treatment of pneumonia during the study period, we only selected the first episode as the index hospitalization. In addition, we limited our study population to those patients who were aged ≥ 65 years old (*n* = 19,682). In this study, we defined subjects who had received statin or ACEI/ARB prescriptions for ≥60 days within 6 months before the index date as statin or ACEI/ARB users in accordance with prior studies [17,18]. In order to isolate the effects of statin or ACEI/ARB use on the risk of pneumonia, we further excluded 401 patients who had received prescriptions for either drug lasting less than 60 days during the 6 months prior to the date of the index hospitalization. As a result, 19,281 patients were included as cases. We assigned the date of their index hospitalization as the index date.

For controls, we randomly selected 19,281 controls to match the cases in terms of sex, age group (65~69, 70~74, 75~79, and ≥80years), and index year from the remaining beneficiaries. We assured that none of the selected controls had received a diagnosis of pneumonia within 2 years before their index date. We also assured that none of the selected controls had received any statin or ACEI/ARB prescriptions within 6 months prior to the index date. While for cases, the year of the index date was the year in which the cases were hospitalized for pneumonia treatment, for controls, the year of the index date was simply a matched year in which controls had a medical utilization. We further assigned their first utilization of medical care occurring in the index year as their index date.

### Outcome measures

This case-control study used the LHID2005 which included the records regarding outpatient services and the relevant prescriptions. We defined those cases who received prescriptions for statin or ACEI/ARB based on the medication records in LHID2005. Additionally, this study only included cases who had received statin or ACEI before the index date.

### Statistical analysis

This study used the SAS system (SAS System for Windows, vers. 8.2, SAS Institute, Cary, NC) to perform all statistical analyses. We used Chi-squared tests to compare differences in sociodemographic characteristics (monthly income, and geographical location and urbanization level of the patient’s residence) and medical comorbidities (diabetes, coronary heart disease, chronic obstructive pulmonary disease, asthma, malignancy, and stroke) between patients who were hospitalized for pneumonia treatment and controls. We used a conditional logistic regression (conditioned on sex, age group, and index year) to compute the odds ratio (OR) and 95% confidence interval (CI) for having previously used statins and/or ACEIs/ARBs between patients who were hospitalized for pneumonia treatment and controls. The conventional *p*<0.05 was used to assess the statistical significance.

## Results

This mean age of the study sample, including 19,281 patients who were hospitalized for pneumonia treatment and 19,281 controls, was 78.5 ± 7.1 years, and respective mean ages were 78.3 and 78.5 years for patients who were hospitalized for pneumonia treatment and the controls (*p* = 0.412). Distributions of sociodemographic characteristics and medical comorbidities between cases and controls are presented in Table 1. After matching for sex, age group, and index year, it shows that patients who were hospitalized for pneumonia treatment were more likely to reside in the northern part of Taiwan and in the most urbanized areas compared to the controls (both *p*<0.001). Furthermore, patients who were hospitalized for pneumonia treatment had higher prevalences of diabetes, coronary heart disease, chronic obstructive pulmonary disease, asthma, malignancy, and stroke than did the controls (all *p*<0.001).

**Table 1:** Demographic characteristics of patients hospitalized for pneumonia and comparison patients.

Variable	Patients with pneumonia (*N* = 19,281)		Comparison patients (*N* = 19,281)		*p* value
	Total no.	%	Total no.	%	
Male	12,441	64.5	12,441	64.5	>0.999
Age (years)					>0.999
65~69	2388	12.4	2388	12.4	
70~74	3284	17.0	3284	17.0	
75~79	4295	22.3	4295	22.3	
≥80	9314	48.3	9314	48.3	
Monthly income ^a^					<0.001
≤ NT$15,840	11,331	58.8	12,030	62.4	
NT$15,841~25,000	6919	35.9	6776	35.1	
≥NT$25,001	1031	5.3	475	2.5	
Geographic region					<0.001
Northern	8365	43.4	7818	40.5	
Central	4863	25.2	4946	25.7	
Southern	5555	28.8	5793	30.1	
Eastern	498	2.6	724	3.7	
Urbanization level					<0.001
1 (highest)	4902	25.4	4175	21.7	
2	4828	25.0	4845	25.1	
3	2977	15.4	2871	14.9	
4	3380	17.5	3720	19.3	
5 (lowest)	3194	16.6	3670	19.0	
Diabetes	6655	34.5	4967	25.8	<0.001
Coronary heart disease	7069	36.7	6058	31.4	<0.001
Chronic Obstructive Pulmonary Disease	1465	7.6	392	2.0	<0.001
Asthma	4501	23.3	2357	12.2	<0.001
Malignancy	1946	10.1	3075	16.0	<0.001
Stroke	8465	43.9	4603	23.9	<0.001

^a^ The average exchange rate in 2010 was US$1.00≈New Taiwan (NT)$30.

Table 2 presents distributions of statin and ACEI/ARB use between patients who were hospitalized for pneumonia treatment and the controls. Of the study sample of 38,562 patients, 1,564 (4.1%) were statin-only users, 15,746 (40.8%) were ACEI/ARB-only users, and 7,934 (20.6%) were statin and ACEI/ARB users. Moreover, we found that there were significant associations of pneumonia hospitalization with statin-only users (*p*<0.001), with ACEI/ARB-only users (*p*<0.001), and with statin and ACEI users (*p*<0.001).

**Table 2:** Prevalence of statin and angiotensin-converting enzyme inhibitor (ACEI)/ angiotensin II receptor blocker (ARB) users in the pneumonia and control groups.

Variable	Patients with pneumonia (*N* = 19,281)	Comparison patients (*N* = 19,281)	*p* value
	*n (column* %)		
Statin-only users			
Yes	496 (2.57)	1068 (5.54)	<0.001
ACEI/ARB-only users			
Yes	8563 (44.41)	7183 (37.25)	<0.001
Statin and ACEI/ARB users			
Yes	3258 (16.90)	4676 (24.25)	<0.001

Table 3 shows the covariate-adjusted ORs for pneumonia hospitalization. After adjusting for monthly income, geographical location and urbanization level of the patient’s residence, diabetes, coronary heart disease, chronic obstructive pulmonary disease, asthma, malignancy, and stroke, the conditional logistic regression analysis (conditioned on sex, age group, and index year) suggested that statin-only users (OR = 0.38, 95% CI = 0.34~0.43), ACEI/ARB-only users (OR = 0.86, 95% CI = 0.82~0.91), and statin and ACEI/ARB users (OR = 0.47, 95% CI = 0.44~0.50) were less likely to have been hospitalized for pneumonia treatment than subjects who had used neither statins nor ACEIs. Furthermore, we found that statin-only users (adjusted OR = 0.44, 95% CI = 0.40~0.50) and statin and ACEI/ARB users (adjusted OR = 0.55, 95% CI = 0.52~0.58) were less likely to have been hospitalized for pneumonia treatment compared to ACEI/ARB-only users. However, statin and ACEI/ARB users (adjusted OR = 1.24, 95% CI = 1.10–1.40) were more likely to have been hospitalized for pneumonia treatment compared to statin-only users.

**Table 3:** Covariate-adjusted odds ratios (ORs) and 95% confidence intervals (CIs) for pneumonia among sampled patients (N = 38,562).

Variable	Hospitalized for pneumonia treatment		
	Adjusted OR ^a^ (95% CI)		
Statin-only users	0.38*** (0.34–0.43)	0.44*** (0.40–0.50)	1.00
ACEI/ARB-only users	0.86*** (0.82–0.91)	1.00	2.25*** (2.01–2.53)
Statin + ACEI/ARB users	0.47*** (0.44–0.50)	0.55*** (0.52–0.58)	1.24*** (1.10–1.40)
Neither statin nor ACEI/ARB users	1.00	1.16*** (1.10–1.22)	2.61*** (2.33–2.94)

Notes: The OR was calculated by a conditional logistic regression which was conditioned on sex and age group

^a^ Adjusted for monthly income, urbanization level, geographic region, stroke, diabetes, coronary heart disease, asthma, chronic obstructive pulmonary disease, and malignancy

*** p<0.001.

## Discussion

We found that both statins and ACEIs/ARBs were significantly associated with a lower risk of being hospitalized for pneumonia in the general population. This finding is consistent with some previous reports. Myles et al. conducted a population-based case-controlled study in the UK and found that both current ACEI users and statin users were associated with reduced risks for community-acquired pneumonia [19]. Our data support that ACEI/ARB users and statin users are at lower risk for pneumonia. Due to the availability and safety profiles of statins and ACEIs/ARBs, the use of these medications may become a possible adjuvant treatment for managing infectious diseases such as pneumonia. It might not be reasonable or cost-effective to routinely prescribe ACEIs/ARBs and statins in the general population only to prevent pneumonia. On the other hand, prescription of these medications under current guideline to treat hypertension and hyperlipidemia should be encouraged and emphasized in general practice.

However, it remains unclear whether initiating these medications at the time of diagnosis is beneficial in reducing the risk of pneumonia. Use of statins and ACEIs/ARBs prior to admission may be considered as a “pre-medications” to condition these patients. In contrast, the data regarding combined use statin/ACEIs/ARBs during the hospitalization for infectious diseases may be seen as a combined treatment to directly modulate the host response to inflammation and infection. Mortensen et al investigated the pneumonia related outcomes in separately outpatient and inpatient use of each of ACEIs/ARBs/statins. They found the uses of statin/ACEI/ARB were associated with improved outcomes of pneumonia except for prior use of ACEI or ARB patients had no reduced mechanical ventilator use [13]. Contradictory, Cheng et al. found concurrent use of statin was associated with better outcome of pneumonia than prior use of statin in a met-analysis [20]. Nevertheless, to evaluate the effects of time initiating statin/ACEI/ARB on the outcomes of pneumonia with a retrospective observational study will face some problems. There were few patients who originally did not use statins/ ACEIs/ARBs prior to admission received these medications during hospitalization for pneumonia in clinical data. This will result in unbalanced distribution during patient inclusion while analyzing the data. Furthermore, the onset of infectious disease is often insidious, therefore, the previous use of statin and ACEI / ARB may actually cover the early phase of infectious disease prior to admission. Therefore, a randomized controlled trial is still necessary and more suitable to investigate the effects of combined statin / ACEI / ARB with anti-infectious agents/antibiotics in treating infectious diseases.

Healthy user bias cannot be excluded in the present observational study. Patients who regularly took statins and/or ACEIs/ARBs to control their disease might have health-promoting behaviors, which would decrease their risk of coming down with pneumonia. In addition, people with health-promoting behaviours might be more likely to seek medical care and be hospitalized than those who do not have these behaviours. De Groot et al. suggested heterogeneity in case-control studies of associations between community-acquired pneumonia and ACEIs/ARBs and statins, which may explain the inconsistent results in that observational study [21]. Moreover, in case-control studies, it is difficult to adjust for bias caused by uncertainty over exposure.

However, we found no significant synergic or additional effects of combining ACEIs/ARBs and statins to prevent hospitalization for pneumonia compared to patients who used only statins. To our best knowledge, this is the first study to compare the effects of combined statin and ACEI/ARB use versus solely statin or ACEI/ARB use in reducing the risk of pneumonia. Although the mechanism of the lack of a response in reducing the risk of pneumonia of combined ACEIs/ARBs and statins is unknown, several potential mechanisms might be considered. First, this finding may imply the mechanisms of anti-infectious and pleiotropic effects of statins and ACEIs/ARBs involve some overlapping cellular pathways. Saturation or an interference effect may have resulted in the poor combined effects of these medications. Second, patients who received combined ACEIs/ARBs/statins may have had more comorbidities than sole users. Interactions of underlying diseases such as hypertension, hyperlipidemia, and cardiovascular diseases contributing to pneumonia should be investigated. These underlying diseases may be associated with a variety degree of endothelial /mitochondrial dysfunctions but it was difficult to fully adjust for this in the present study. Interestingly, Hoang et al. performed a meta-analysis based on randomized control trials to investigate the effects of statin use combined with use of ACEIs and ARBs on cardiovascular disease prevention. They noted that the effect of ACEIs/ARBs in reducing cardiovascular morality was attenuated after adjusting for statin use [1]. This finding supports that the combined use of statins and ACEIs/ARBs might have interactions that cannot be neglected in reducing pneumonia, which needs to be further investigated.

A meta-analysis of observational studies including cohort and case-control studies reported that mortality from pneumonia showed a benefit (adjusted OR = 0.89, 95% CI, 0.81~0.97, Number need to treat inpatients = 230) in statin users [22]. Furthermore, current statin users might have better outcomes than recent or past statins users. This study supported patients who received statin therapy having less mortality from pneumonia [22]. However, some authors suggested that the beneficial effects of statins on influenza-related adverse outcomes may be explained by a healthy user bias [23]. Polgreen et al. found that the use of statins was associated with a reduction in pneumonia. However, they found that for patients with acute myocardial infarction, the protective effect of statins against pneumonia may have been associated with non-randomized allocations [24]. Contradictorily, one cohort study showed that hospitalized patients treated for infection and with statin exposure were not associated with any outcome [25]. Patel et al. showed that patients received the atorvastatin 40mg daily had a significantly lower rate to develop severe sepsis compared to placebo (4% vs. 24% p = 0.007) in a randomized sham controlled trial [34]. On the other hand, results from other prospective randomized controlled studies found insufficient evidence regarding the beneficial effects of statins in protecting against infection, although they usually had small sample sizes [26,27]. Viasus et al. found that the use of simvastatin, 20 mg daily for 4 days since hospital admission, did not reduce the time to clinical stability in hospitalized patients with community-acquired pneumonia [26]. Another double-blind randomized clinical trial of 149 patients undergoing neurosurgeries showed that patients with lovastatin, 1 day before the operation and 3 days after surgery, had fewer but not significant infectious events compared to controls [27]. Furthermore, large randomized controlled studies showed there is no improvement of outcomes in the acute respiratory distress and ventilator associated pneumonia in ICU setting with treatment of statins [28–30]. Unable to show the protective effects of statin from these studies may be associated with the dose and timing of medications. Further clinical trials to investigate these specific issues may be still needed.

The mechanisms in protective effects of statins / ACEIs /ARBs might open a reasonable way to develop clinical treatment to a variety of infectious disease. Treating the host response provides important strategies to decrease systemic inflammation induced organ damage and endothelial dysfunction may be important to improve outcomes as well [8]. Therefore, it has been suggested that develop adequate clinical strategies with combining these generic medications might be a potential achievable way in treating other infectiously disease such as influenza and emerging infectiously diseases [7].

The present study was a large-scale cohort study and provides the strength of sufficient statistical power. However, there were several limitations in the present study that should be addressed. First, the validity of medical coding of the present study is an intrinsic problem of medical registry-based studies. Nevertheless, the diagnostic accuracy of diseases and outcomes in the NHIRD was reported to be acceptable [20,31]. Secondly, some variables were undetermined in the database, such as the body-mass index, cigarette smoking, and physical activity. According to prior literature, patients’ body-mass index and smoking status are potential risk factors for pneumonia [32–35]. These factors may affect the risk of contracting pneumonia and influence the following hospitalization outcomes. For example, Hemilä investigated the protective effects of Vitamin E against pneumonia and found the effectiveness of Vitamin E were modified by cigarette smoking and physical exercise [36]. Third, the database which used in this study did not provide the information regarding lab data and medical chart. Therefore, we could not assure whether the risk of pneumonia was due to poorly controlled comorbidities (such as poorly controlled diabetes or COPD) in this study. Fourth, the list for potential medications affecting inflammation and immunological profiles is too long, so we did not analyze the effects of several medications which may affect inflammation and outcomes such as other antihypertensive, other lipid-lowering medications, metformin, aspirin, macrolides, etc. in the present study. Fifth, the primary goal in present study is to investigate the protective effects of ACEIs/ARBs/statins on risk for pneumonia. Present study did not analyze the pneumonia associated outcomes such as morality, or respiratory failure with ventilator uses. The analysis regarding outcomes needs to consider more additional clinical variables and different statistical modelling which is beyond the scope of present study. Further study should be conducted in analyzing this important issue. Finally, the dose-dependent response and types of ACEIs/ARBs/statins should be considered when analyzing effects of statins and ACEIs/ARBs. A nested case-control analysis of a retrospective, population-based cohort of statin users showed that high-potency statins were not associated with a decreased risk of community-acquired pneumonia compared to low-potency statins [37].

We found that both statins and ACEIs/ARBs were associated with a lower risk of pneumonia occurrence in a large population study. However, combined use of statins and ACEIs/ARBs did not exhibit a synergistic effect in protecting against pneumonia.
